# An Ensemble Method for Predicting Subnuclear Localizations from Primary Protein Structures

**DOI:** 10.1371/journal.pone.0057225

**Published:** 2013-02-27

**Authors:** Guo Sheng Han, Zu Guo Yu, Vo Anh, Anaththa P. D. Krishnajith, Yu-Chu Tian

**Affiliations:** 1 School of Mathematics and Computational Science, Xiangtan University, Xiangtan City, Hunan, China; 2 School of Mathematical Sciences, Queensland University of Technology, Brisbane, Queensland, Australia; 3 School of Electrical Engineering and Computer Science, Queensland University of Technology, Brisbane, Queensland, Australia; University of Alberta, Canada

## Abstract

**Background:**

Predicting protein subnuclear localization is a challenging problem. Some previous works based on non-sequence information including Gene Ontology annotations and kernel fusion have respective limitations. The aim of this work is twofold: one is to propose a novel individual feature extraction method; another is to develop an ensemble method to improve prediction performance using comprehensive information represented in the form of high dimensional feature vector obtained by 11 feature extraction methods.

**Methodology/Principal Findings:**

A novel two-stage multiclass support vector machine is proposed to predict protein subnuclear localizations. It only considers those feature extraction methods based on amino acid classifications and physicochemical properties. In order to speed up our system, an automatic search method for the kernel parameter is used. The prediction performance of our method is evaluated on four datasets: Lei dataset, multi-localization dataset, SNL9 dataset and a new independent dataset. The overall accuracy of prediction for 6 localizations on Lei dataset is 75.2% and that for 9 localizations on SNL9 dataset is 72.1% in the leave-one-out cross validation, 71.7% for the multi-localization dataset and 69.8% for the new independent dataset, respectively. Comparisons with those existing methods show that our method performs better for both single-localization and multi-localization proteins and achieves more balanced sensitivities and specificities on large-size and small-size subcellular localizations. The overall accuracy improvements are 4.0% and 4.7% for single-localization proteins and 6.5% for multi-localization proteins. The reliability and stability of our classification model are further confirmed by permutation analysis.

**Conclusions:**

It can be concluded that our method is effective and valuable for predicting protein subnuclear localizations. A web server has been designed to implement the proposed method. It is freely available at http://bioinformatics.awowshop.com/snlpred_page.php.

## Introduction

The cell nucleus is the most important organelle within a cell. It directs cell reproduction, controls cell differentiation and regulates cell metabolic activities [1]–[3]. The nucleus can be further subdivided into subnuclear localizations, such as PML body, nuclear lamina, nucleoplasm, and so on. The subcellular localizations of proteins are closely related with their functions. A mis-localization of proteins can lead to protein malfunction and further cause both human genetic disease and cancer [4]. At the subnuclear level, elucidation of localizations can reveal not only the molecular function of proteins but also in-depth insight on their biological pathways [1], [3].

It is time-consuming and costly to find subnuclear localizations only by conducting various experiments, such as cell fractionation, electron microscopy and fluorescence microscopy [5]. On the other hand, the large gap between the number of protein sequences generated in the post-genomic era and the number of completely characterized proteins has called for the development of fast computational methods to complement experimental methods in finding localizations.

There have been various methods for predicting protein subcellular localizations based on sequence information [2], [6]–[17] as well as non-sequence information, such as function domain [18], gene ontology [19]–[22], evolutionary information [20], [23]–[27], and protein-protein interaction [28]. Some methods predict subcellular localizations at specific genomic level [16], [20], [24], [29], [30]. These methods did not provide information on subnuclear localizations.

So far, a few methods have been reported for predicting protein subnuclear localizations [1], [2], [21], [25]–[27]; however their prediction accuracies are relatively poor for small size localizations. The prediction of localizations at the subnuclear level is more challenging than that at the subcellular level due to three factors [31]–[33]: the nucleus is more compact and complicated as compared to other cell compartments [32]; protein complexes within the cell nucleus can alter their compartments during different phases of the cell cycle [33]; and proteins within the cell nucleus face no apparent physical barrier like a membrane [31]. In the face of these difficulties, we believe that diverse information is required to solve this problem. Feature extraction methods from different sources can complement each other in capturing valuable information, and prediction accuracy can be enhanced through effectively combining those feature extraction methods.

In this paper, we design a novel two-stage multiclass support vector machine (MSVM) in combination with a two-step optimal feature selection process for successfully predicting protein subnuclear localizations. The process incorporates various features extracted from amino acid classifications-based methods including local amino acid composition (LAAC) [11], local dipeptide composition (LDC) [11], global descriptor (GD) [34], Lempel-Ziv complexity (LZC) [35], and those extracted from physicochemical properties-based methods including autocorrelation descriptor (AD) [36], sequence-order descriptor (SD) [36], [37], autocovariance method (AC) [38]–[40], physicochemical property distribution descriptor (PPDD) [41], recurrence quantification analysis (RQA) [42], discrete wavelet transform (DWT) [43] and Hilbert-Huang transform (HHT) [44], [45]. If each protein is represented by all these obtained features, the dimension of the feature vector will be too high. In order to reduce computation complexity and feature abundance, we propose a two-step optimal feature selection process to find the optimal feature subset for each binary classification, which is based on the maximum relevance and minimum redundancy (mRMR) feature prioritization method [46]. We use the one-against-one (OAO) strategy to solve the multiclass problem: for a *k* classification problem, 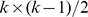 classifiers will be constructed. In our system, these classifiers are all constructed using support vector machine with probability output. After this, the high-dimensional feature vector of each protein is converted into a probability vector with 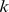 dimensions. At the second stage, conventional MSVM is used to construct the final models.

## Results and Discussion

### Data Sets

We chose two datasets, Lei dataset [1] and SNL9 dataset [26], to evaluate the performance of our method in comparison with previous methods. Lei dataset was extracted from the Nuclear Protein Database (NPD) [47] and is non-redundant with less than 50% sequence identity. It consists of 504 proteins divided into 6 subnuclear localizations: 38 belong to PML body, 55 to nuclear lamina, 56 to nuclear splicing speckles, 61 to chromatin, 75 to nucleoplasm, and 219 to nucleolus. Each of these proteins belongs to a single localization. This data set is unbalanced because the size of the largest localization is 219, whereas the smallest is just 38. The SNL9 dataset was collected from Swiss-Prot (version 52.0 released on 6 May 2007) at http://www.ebi.ac.uk/swissprot/by following a strict five-step filter procedure. The details about this procedure can be found in [26]. The final data set contains 714 proteins, of which 99 belong to chromatin, 22 to heterochromatin, 61 to nuclear envelope, 29 to nuclear matrix, 79 to nuclear pore complex, 67 to nuclear speckle, 307 to nucleolus, 37 to nucleoplasm and 13 to nuclear PML body. All sequences have <80% sequence identity.

In order to estimate the effectiveness of our prediction method, two independent testing sets are used. One consists of 92 multi-localization proteins, which was also constructed by Lei et al. [1]. Another is constructed from SNL9 dataset. We only select 5 types which are in Lei dataset because this dataset does not contain nuclear lamina. Then, we filter out those which have larger than 30% sequence identity with any other in Lei dataset. The final dataset includes 328 proteins: 8 belong to PML body, 36 to nuclear splicing speckles, 77 to chromatin, 25 to nucleoplasm, and 182 to nucleolus.

### Amino Acid Classification

To capture more contextual information, the LAAC [11], LDC [11], GD [34] and LZC [35] methods consider different amino acid classification approaches. Some of these approaches [36], [48]–[53] are listed in Table 1.

**Table 1:** Amino acid classifications.

Method	Number	Amino acid classification	Reference
HP	2	(ALIMFPWV) (DENCQGSTYRHK)	[48]
DHP	4	(ALVIFWMP) (STYCNGQ) (KRH) (DE)	[49]
7-Cat	7	(AGV) (ILFP) (YMTS) (HNQW) (RK) (DE) C	[50]
20-Cat	20	A G V I L F P Y M T S H N Q W R K D E C	-
ms	6	(AVLIMC) (WYHF) (TQSN) (RK) (ED) (GP)	[51]
lesk	6	(AST) (CVILWYMPF) (HQN) (RK) (ED) G	[51]
F-Ic4	7	(AWM) (GST) (HPY) (CVIFL) (DNQ) (ER) K	[51]
F-Ic2	9	(AWM) (GS) (HPY) (CVI) (FL) (DNQ) (ER) K T	[51]
F-IIIc4	9	(ACV) (HPL) (DQ) S (ERGN) F (IMT) (KW) Y	[51]
F-Vc4	8	(AWHC) G (LEPV) (KYMT) (IN) Q D S	[51]
Murphy8	8	(LVMIC) (AG) (ST) P (FYW) (DENQ) (KR) H	[52]
Murphy15	15	(LVIM) C A G S T P (FY) W E D N Q (KR) H	[52]
Letter12	12	(LVIM) C (AG) (ST) P (FY) W (ED) N Q (KR) H	[53]
Hydrophobicity	3	(RKEDQN) (GASTPHY) (CLVIMFW)	[36]
Normalized van der Waals	3	(GASTPD) (NVEQIL) (MHKFRYW)	[36]
Polarity	3	(LIFWCMVY) (PATGS) (HQRKNED)	[36]
Polarizability	3	(GASDT) (CPNVEQIL) (KMHFRYW)	[36]
Charge	3	(KR) (ANCQGHILMFPSTWYV) (DE)	[36]
Secondary structure	3	(EALMQKRH) (VIYCWFT) (GNPSD)	[36]
Solvent accessibility	3	(ALFCGIVW) (PKQEND) (MPSTHY)	[36]

### Physicochemical Properties

In order to capture as much information of protein sequences as possible, a variety of physicochemical properties are used in the procedure of feature extraction. All physicochemical properties used can be found in the Amino Acid index (AAindex) database [54], which store physicochemical or biochemical properties of amino acids or pair of amino acids. The latest version of the database (version 9) is separated into three parts: AAindex1, AAindex2 and AAindex3. AAindex1 has 544 properties associated with each of the 20 amino acids, AAindex2 contains 94 amino acid substitution matrices, and AAindex3 contains 47 amino acid contact potential matrices. For the purpose of amino acid sequence transformation, we only considered the 544 amino acid properties (i.e., indices in AAindex1). Of the 544 indices, 13 have incomplete data or an over-representation of zeros, hence were removed. Thus 531 indices were evaluated for potential use in the procedure of feature extraction. In particular, in the AD method we chose the 30 physicochemical properties of amino acids as in [55], which are listed in Table 2.

**Table 2:** 30 physicochemical properties of amino acids selected from AAindex database.

AAindex	Physicochemical property	Range of property
BULH740101	Transfer free energy to surface	[−2.46 0.16]
BULH740102	Apparent partial specific volume	[0.558 0.842]
PONP800106	Surrounding hydrophobicity in turn	[10.53 13.86]
PONP800104	Surrounding hydrophobicity in alpha-helix	[10.98 14.08]
PONP800105	Surrounding hydrophobicity in beta-sheet	[11.79 16.49]
PONP800106	Surrounding hydrophobicity in turn	[9.93 15.00]
MANP780101	Average surrounding hydrophobicity	[11.36 15.71]
EISD840101	Consensus normalized hydrophobicity scale	[−1.76 0.73]
JOND750101	Hydrophobicity	[0.00 3.15]
HOPT810101	Hydrophilicity value	[−3.4 3.00]
PARJ860101	HPLC parameter	[−10.00 10.00]
JANJ780101	Average accessible surface area	[22.8 103.0]
PONP800107	Accessibility reduction ratio	[2.12 7.69]
CHOC760102	Residue accessible surface area in folded protein	[18 97]
ROSG850101	Mean area buried on transfer	[62.9 224.6]
ROSG850102	Mean fractional area loss	[0.52 0.91]
BHAR880101	Average flexibility indices	[0.295 0.544]
KARP850101	Flexibility parameter for no rigid neighbors	[0.925 1.169]
KARP850102	Flexibility parameter for one rigid neighbor	[0.862 1.085]
KARP850103	Flexibility parameter for two rigid neighbors	[0.803 1.057]
JANJ780102	Percentage of buried residues	[3 74]
JANJ780103	Percentage of exposed residues	[5 85]
LEVM780101	Normalized frequency of alpha-helix, with weights	[0.90 1.47]
LEVM780102	Normalized frequency of beta-sheet, with weights	[0.72 1.49]
LEVM780103	Normalized frequency of reverse turn, with weights	[0.41 1.91]
GRAR740102	Polarity	[4.9 13.0]
GRAR740103	Volume	[3 170]
MCMT640101	Refractivity	[0.00 42.35]
PONP800108	Average number of surrounding residues	[4.88 7.86]
KYTJ820101	Hydropathy index	[−4.5 4.5]

### System Construction

#### Support vector machine

In 1995, Vapnik [56] introduced the support vector machine (SVM) method to solve the binary classification problem. In order to solve a multiclass classification problem, such as the prediction of protein subnuclear localizations, the method must be extended. There are three notable extension strategies: one-against-all, one-against-one and directed acyclic graph SVM (DAGSVM) [57]. In this paper, we adopted the one-against-one strategy. For a 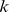 classification problem, the SVM designed by the one-against-one strategy constructs 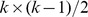 classifiers, each of which is trained on data from two different classes. The optimal complexity parameter 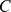 in the SVM classifier is fixed by grid search. Throughout, the radial basis kernel function (RBF) is used and the corresponding kernel parameter 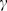 can be determined by grid search or automatic methods [58], [59]. We select the method GFO for the supervised case proposed in [59] due to its simplicity. In GFO, the optimal kernel parameter 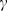 is approximated by the mathematical expectation of distances between data points.

Furthermore, we used a weighting scheme as in [60] for each class in order to reduce the effect of over-prediction when using unbalanced training data sets. The weighting scheme assigns weight 1.0 to the largest class and higher weights to the remaining classes. The weights of these classes are simply calculated by dividing the size of the largest class by that of each smaller class.

#### Two-step optimal feature selection

After running each feature extraction method, all primary protein structures with different length are converted into numerical feature vectors with the same dimension. In order to reduce feature abundance and computation complexity, we propose a two-step optimal feature selection process by using an incremental feature selection (IFS) method [61].

The IFS is based on the mRMR method originally proposed by [46] for analyzing microarray data. The detailed information about the mRMR and IFS methods can be found in [46], [61], respectively. In the first step, we consider each feature extraction method separately and construct corresponding models for each binary classification. Supposing that the number of feature extraction methods used is 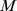, there are 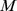 optimal feature subsets constructed for each binary classification in this step. In the second step, for each binary classification, we extract the final optimal feature subset on the union of 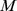 optimal feature subsets obtained in the first step. We simultaneously find the optimal feature subset and the SVM parameters 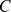 and 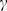 for each binary classification using 5-fold cross validation on the training set for each turn in the leave-one-out cross validation process.

#### Two-stage support vector machine

**Figure 1:**
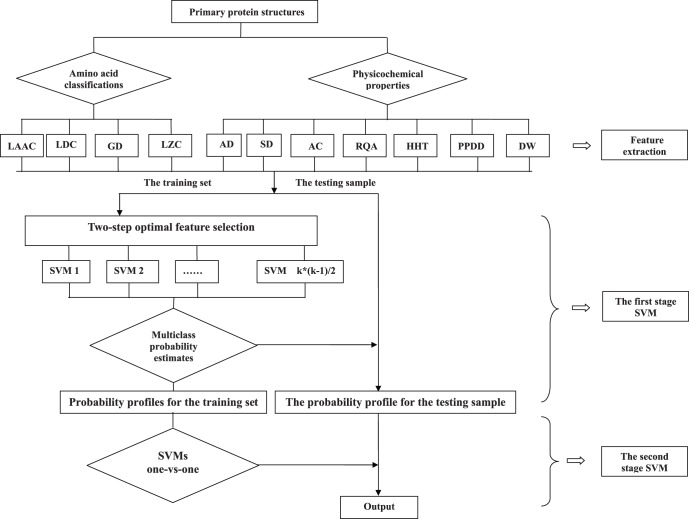
The architecture of our method.

Finally, we construct a novel two-stage support vector machine to predict protein subnuclear localizations. In the first stage, 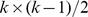 binary classifiers with probability estimates are constructed based on the two-step optimal feature selection procedure for each turn in the leave-one-out cross validation process. All optimal feature subsets and SVM parameters for 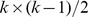 binary classifiers are simultaneously obtained by the two-step optimal feature selection procedure. We use LIBSVM for probability estimation as in [62]. After this, each primary protein structure is represented by a *k*-dimensional numerical vector, each element of which is the probability of the corresponding class to be predicted. The outputs of this stage are used as inputs for the next stage. In the second stage, we use conventional multiclass SVMs to predict protein subnuclear localizations. Here we use LIBSVM [62] to implement SVMs. The complete flow chart of our method is shown in Figure 1. Note that if the leave-one-out cross validation is chosen to test this two-stage SVM, different two-stage SVM is constructed for each turn the leave-one-out cross validation.

### Performance Evaluation

In statistical prediction, three validation tests are often used to evaluate the prediction performance: independent dataset test, sub-sampling test and jackknife test [63]. We adopted the jackknife test in this paper to make fair comparison with existing methods. That is, each protein sequence in the samples is singled out in turn as a test sample and the remaining protein sequences are used as training samples. In this sense, the jackknife test is also known as the leave-one-out test.

The overall prediction accuracy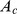, individual sensitivity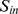, individual specificity 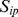
and Matthew’s correlation coefficient 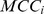 are used to evaluate the prediction performance of our work. Their definitions are as follows:
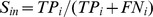


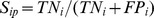


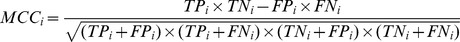


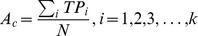
where true positives *TP* = number of positive events that are correctly predicted; true negatives *TN* = number of negative events that are correctly predicted; false positives *FP* = number of negative events that are incorrectly predicted to be positive; false negatives *FN* = number of subjects that are predicted to be negative despite they are positive; *k* = number of classes.

To further evaluate the performance of our method, we also use the receiver operating characteristic (ROC) curve [64], which is probably one of the most robust approaches for classifier evaluation. The ROC curve is obtained by plotting true positive rate (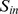) on the y-axis against the false positive rate (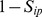) on the x-axis. The area under the ROC curve (AUC) [65] can be used as a reliable measure for the prediction performance. The case that maximum value of AUC equals to 1 means a perfect prediction. A random guess receives an AUC value close to 0.5.

### Comparison of Feature Extraction Methods: Grid Search vs Automatic Search

First, we observed each feature extraction method separately to see which method is more effective. The same leave-one-out cross validation process as [1] is used to evaluate each feature extraction method and their combinations on Lei benchmark dataset. For details, during the training process, each protein is selected as the test sample in turn and the remaining ones constitute the training set. We used a grid search approach to find optimal feature subsets and optimize the SVM parameter 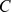 using 5-fold cross validation on the training set for all binary classification models. For the SVM parameter 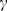, we use two kinds of methods to find the optimal value: grid search and GFO [59]. It is found that the number of elements of the optimal feature subset for each binary classification is generally less than 300. So we chose the top-rank 300 features as the upper bound for optimal feature subset search. The top-rank 10 features are used as an initial feature subset. The size of the feature subset is increased by 10, obtaining 10, 20, 30,..., 300 features. At each size, we searched a pair (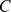,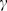) with the best 5-fold cross validation (e.g. log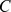 = −5, −3, −1,..., 15; log 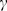 = −15, −13, −11,..., 3). From this process, each binary classification model corresponds to an optimal feature subset and a parameter pair (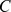,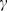). Thus we can construct all binary classification models and make preparation for training the second stage model. The training method for the second stage model is identical to the first stage except that it does not need feature selection. The final prediction system was constructed as follows: the entire Lei dataset of proteins is used as a training set; the optimal feature subsets for each binary classification are taken as the union of all optimal feature subsets obtained from the leave-one-out cross validation; and the optimal value for each parameter of the SVMs for the training set was taken as the average value of the optimal parameters obtained from the leave-one-out cross validation. And then the final system is tested on the multi-localization dataset and the new independent testing set. Note that all parameters of the final system including optimal features and SVM parameters are not re-paramiterized to apply on the independent datasets.

The overall prediction accuracies for all feature extraction methods on Lei data set and the new independent dataset are listed in Table 3. We also combined the feature extraction methods LAAC, LDC, GD, LZC, AD, SD and AC as one method, named *Combination1*, in order to balance the number of features used in the methods. In the following, the values on the new independent dataset are shown in the parentheses From Table 3, as far as the individual feature extraction method is concerned, broadly speaking, the HHT method is the best. Its prediction accuracy is 63.49% (65.87%), only worse than the accuracy of 69.84% (70.83%) for *Combination1*. Of particular interest, HHT outperforms DWT (57.54 and 56.15%), implying that HHT is more effective. Note that HHT and DWT are both time-frequency analysis methods and use similar definitions of statistical features. Finally, we evaluate the performance of the combination of all feature extraction methods, named *Combination2*. As shown in Table 3, *Combination2* achieves the overall accuracy of 77.8% (75.2%) for single-localization proteins, with accuracy increase against individual methods between 7.9% (4.4%) and 24.2% (22.2%).

**Table 3:** Comparison of the overall prediction accuracy between different feature extraction methods.

Feature extraction method	Grid search		GFO	
	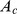 (%)	CPU time (hr)	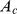 (%)	CPU time (hr)
Combination1	69.84 (59.76)	2.704	70.83 (62.50)	0.406
RQA	53.57 (45.12)	2.174	52.98 (44.82)	0.413
HHT	63.49 (60.37)	2.336	65.87 (64.63)	0.427
PPDD	56.55 (53.35)	2.213	58.53 (59.15)	0.414
DWT	57.54(52.74)	2.035	56.15 (50.91)	0.402
Combination2	77.78 (70.12)	11.056	75.20 (69.82)	2.303

Note: the values on the new independent dataset are shown in the parentheses.

We can also see from Table 3 that it takes far less CPU time to train the models using GFO comparison with those using grid search. Note that all experiments on the same PC (CPU: Intel Core2 Duo T7700, 2.4 GHz; RAM: 3 GB). In view of this reason, we propose the model using GFO as the system model although its OAs are 2.6% lower than that using grid search.

**Figure 2:**
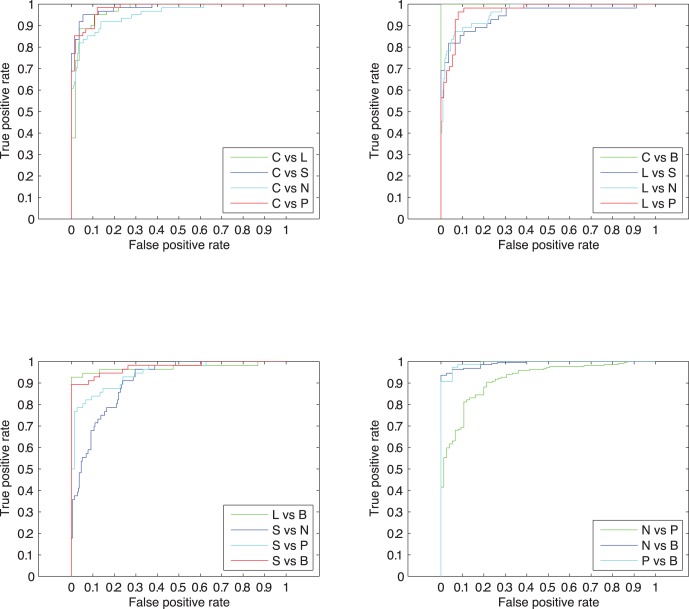
The ROC curves for all binary classifications. The upper letters B, L, S, C, P and N correspond to six subnuclear locations, PML body, nuclear lamina, nuclear speckles, chromatin, nucleoplasm and nucleolus, respectively.

In addition, we also plot the ROC curves for each binary classification in the final prediction system. The ROC curves are shown in Figure 2. All the AUC values for these curves are over 0.9, which indicates that our predictions are satisfactory for all binary classifications. One can see the binary classification for nuclear speckles and nucleolus is the worst one, which degrades the system performance.

### Comparison with the Existing Methods

A comparison of the performance of our method (*Combination2* ) against other existing methods on Lei dataset is illustrated in Table 4, where better results are highlighted in bold. It is seen that *Combination2* achieves an overall accuracy of 77.8% (75.2%) for single-localization proteins against 50.0% of SVM Ensemble [1], against 66.5% of the GO-AA [21]. The measures *Sn*, *Sp* and *MCC* reveal that *Combination2* is far better than SVM Ensemble on all subnuclear localizations, better than GO-AA on most subnuclear localizations except *Nuclear Speckles and Nuclear Lamina.* Note that SVM Ensemble and GO-AA did not give the results on the measure *Sp*. The measures *Sn*, *Sp* and *MCC* reveal that *Combination2* is better than SpectrumKernel on most subnuclear localizations except *Nuclear Speckles* and *Nucleolus*.

**Table 4:** Performance comparison on Lei’s benchmark data set.

Subnuclear localization	size	SVMensemble [1]		Go-AA [21]		SpectrumKernel [2]			Ourmethod		
		*S_n_*	MCC	*S_n_*	MCC	*S_p_*	*S_n_*	MCC	*S_p_*	*S_n_*	MCC
PML Body	38	29.0	0.172	34.2	0.253	11.1	10.5	0.046	86.1(85.3)	**55.3** (52.6)	**0.298**(0.273)
Nuclear Lamina	55	43.6	0.338	63.6	0.578	51.9	50.9	0.461	91.0 (91.9)	69.1 (**70.9**)	0.534(**0.572**)
Nuclear Speckles	56	35.7	0.363	62.5	0.607	86.7	**69.6**	**0.754**	91.8 (91.1)	62.5 (53.6)	0.503(0.460)
Chromatin	61	19.7	0.260	60.7	0.518	64.3	59.0	0.570	93.1(93.1)	**73.8** (65.6)	**0.640**(0.572)
Nucleoplasm	75	22.7	0.206	56.0	0.504	52.6	54.7	0.465	90.8 (89.2)	64.0 (**66.7**)	**0.526**(0.520)
Nucleolus	219	76.7	0.367	79.0	0.656	89.8	**96.4**	**0.880**	78.6 (75.9)	93.6 (91.3)	0.726(0.570)
OA for single-localization		50.0		66.5			71.2			77.8(75.2)	
OA for multi-localization		65.2		65.2			-			**76.1**(71.7)	

Note: the values about models using GFO are shown in the parentheses.

In order to make fair and reasonable comparison with the SpectrumKernel method [2], we test our method using 5-fold cross validation on Lei dataset. Its accuracies are 79.0% and 77.6%, which are both obviously higher than 71.2% of the SpectrumKernel method.

As shown in Table 4, *Combination2* achieves better performance on most small-size subnuclear localizations except *Nuclear Speckles*. The performance of our method on large-size subnuclear localizations *Nucleolus* is worse than SpectrumKernel; however it also achieves 93.6% (91.3%) for *Sn*, which outperforms SVM Ensemble (76.7%) and GO-AA (79.0%). Overall, the results show that our method has good generalization abilities in predicting subnuclear localizations regardless of the size of subnuclear localizations.

In order to evaluate the performance of our method for multi-localization proteins, we use the same criterion as in [1], [21], [66]. For a protein with multi-localization, if one of the locations is predicted true, then the entire prediction is considered correct. For the independent set of multi-localization proteins, the overall accuracy of *Combination2* is 76.1% (71.7%), 11.1% (6.7) higher than SVM Ensemble [1] and GO-AA [21]. The result reveals that a combination of feature extraction methods integrates more effectively information of the protein sequence to increase the prediction accuracy.

Furthermore, comparing with GO-AA, our method only uses information on amino acids of the protein sequence, and do not use non-sequence information such as GO annotation, evolutionary information (e.g. PSI-BLAST profile), protein-protein interaction and so on, which makes our method more general since the PSI-BLAST profile is difficult to obtain and GO annotation and protein-protein interaction may be missing for some proteins. In addition, SpectrumKernel is based on kernel fusion, which is computationally more intensive than sequence-based methods and is also time consuming for training on a novel query sequence.

Furthermore, in order to make fair and reasonable comparison with Nuc-Ploc [26], we test our method using leave-one-out cross validation on SNL9 dataset. A web-server was designed in Nuc-Ploc [26] by fusing PseAA composition and PsePSSM. The detailed comparison results between our method and Nuc-Ploc are listed in Table 5. As shown in Table 5, the overall accuracy of prediction for 9 localizations is 72.1% in the leave-one-out cross validation on SNL9 dataset, which is about 4.7% higher than the overall accuracy obtained by Nuc-Ploc [26]. All MCCs of our method are higher than Nuc-Ploc except for heterochromatin.

**Table 5:** Performance comparison on SNL9 benchmark data set.

Subnuclear localization	Size	MCC	
		Nuc-Ploc	Our method
Chromatin	99	0.60	0.64
Heterochromatin	22	0.52	0.27
Nuclear envelope	61	0.53	0.58
Nuclear matrix	29	0.52	0.56
Nuclear pore complex	79	0.70	0.70
Nuclear speckle	67	0.43	0.62
Nucleolus	307	0.57	0.69
Nucleoplasm	37	0.31	0.55
Nuclear PML body	13	0.32	0.43
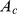 (%)		67.4%	72.1%

Note: MCCs and 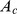 about Nuc-Ploc are obtained directly from the original paper [26].

### Analysis of Feature Contribution

In order to observe the contribution of the individual feature extraction method to the overall prediction accuracy, we test some possible combinations of feature extraction methods. Here, we only report the second best combination for models using grid search and GFO, respectively. For grid search, Combination1+HHT+DWT+PPDD is the second best combination, whose OA are 75.00% and 64.6% on Lei dataset and the new independent dataset. For GFO, Combination1+HHT +PPDD is the second best combination, whose OA are 72.02% and 64.0% on Lei dataset and the new independent dataset.

Moreover, the paired t-test is applied to the MCC values of *Combination2* and other individual methods to evaluate their differences on the new independent dataset. The resulting P-values are reported in Table 6. We can see that the P-values are smaller than 0.05 for all individual methods, indicating that *Combination2* has made statistically significant improvements over any other individual method for the subnuclear localization prediction.

**Table 6:** Comparisons of *Combination2* with the individual method on the new independent dataset.

Methods	Grid search	GFO
	P-values	P-values
Combination1	0.022	0.028
RQA	4.461e−4	3.494e−4
HHT	0.037	0.025
PPDD	0.005	0.004
DWT	0.003	0.001

### Comparison with Other Popular Classifiers

We will also compare our two-stage SVM with Random Forest (RF) classifier [67] as well as traditional “one-stage” SVM [62]. RF consists of a number of unpruned decision trees and is widely used for classification and regression, especially for so-called "small n, large p" problems [67]. It has two advantages: interpretable classification rules and measure information about the importance of features. Here, we use a Matlab package for implementing the RF algorithm [68]. Two parameters, number of trees to grow *ntree* and number of variables randomly sampled as candidates at each split *mtry* are optimized using a grid search approach. During the grid search, the values of *ntree = *500∶500:2000 and *mtry* = (default value) are optimized based on 5-fold cross-validation on Lei dataset. The new independent test set is used to test the final model. For the traditional “one-stage” SVM, we use the same optimization process as two-stage SVM with GFO. In order to investigate the effects of weight strategy on the results, the RF and traditional “one-stage” SVM are divided into two versions: with weight and without weight. All results are illustrated in Table 7. Overall, the traditional “one-stage” SVM is a little better than RF. But, their results are all below 60%, which are much worse than those of two-stage SVM. For individual methods, *Combination1* and HHT are still better than the others. All models using weight strategy demonstrate better or similar results compared with those without using weight strategy.

**Table 7:** Comparisons with other popular classifiers on the new independent dataset.

Methods	TraditionalSVM ( 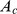 (%))		RandomForest ( 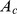 (%))	
	weight	without weight	weight	without weight
Combination1	59.45	57.62	58.54	57.32
RQA	45.73	45.73	45.73	44.82
HHT	59.76	56.10	57.93	56.10
PPDD	58.54	57.93	55.49	55.18
DWT	57.62	55.49	52.74	51.52
Combination2	66.16	64.63	64.02	63.11

In order to evaluate the effectiveness of our two-stage SVM method, we make comparison with another two-stage SVM method used in [11] on Lei dataset and SNL9 dataset. Although a few two-stage SVM methods [69]–[71] have been proposed, they are designed specially for site prediction. In [11], each feature extraction method is viewed as an individual module and each amino acids sequence is transformed into a probability vector in each individual module; the concatenation of these probability vectors output from all modules in the first stage is the input of the second stage. The overall accuracies are 54.17% and 58.12% in the leave-one-out cross validation on Lei dataset and SNL9 dataset respectively, which are obviously lower than corresponding accuracies obtained by our method.

### Assessment of the Reliability of Classification Models by Permutation Analysis

**Figure 3:**
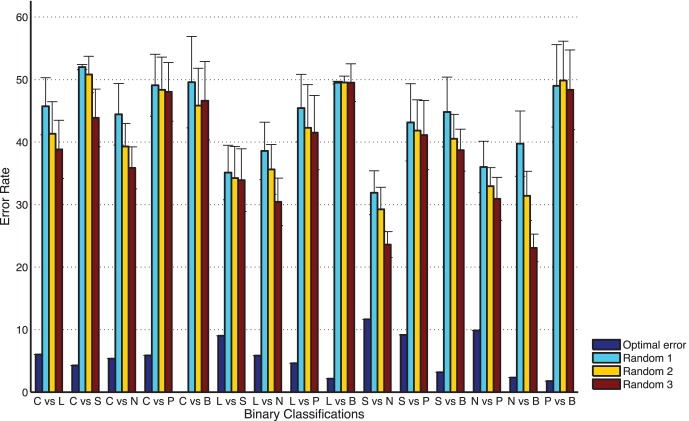
Comparisons of error rate (percentage of misclassified samples) over 50 runs of randomization analysis. Random 1: selecting randomly features subsets from original features, whose size is one-forth of the number of optimal features; Random 2: one half of the number of optimal features; Random 3: equal to the number of optimal features.

In order to evaluate the effectiveness of two-step optimal feature selection method, two kinds of randomization studies were performed for each binary classification. The two kinds of randomization studies are: given the number *K*, randomly select *K* features from original features (case 1) or suboptimal features (case 2) of the samples from two different subnuclear locations, while keeping the class memberships unchanged. Then the newly generated feature set is analyzed by using the same five-fold cross validation as applied before to the original feature set. Here, the given numbers of features *K* are set as one forth, half or all of the number of optimal features. This procedure for case 1 is carried out 50 rounds and the error rates (±standard deviation) over 50 permutations are shown in Figures 3, and compared with the minimum error rates obtained from optimal features. For case 2, similar results are obtained. In each case, the estimated error rate obtained by optimal features is significantly lower than that obtained by the randomization study. Especially, the misclassification error rates obtained by using features selected randomly from suboptimal features are also much lower than that estimated by using those from the original features. If we do these two randomization analysis on the whole original feature set 50 times, overall error rates on average are 63.6% (±4.6%) and 45.5% (±2.4%), which are both significantly higher than the error rate 21.2% obtained by optimal features. Therefore, it can be concluded that two-step optimal feature selection method is effective and reliable.

**Figure 4:**
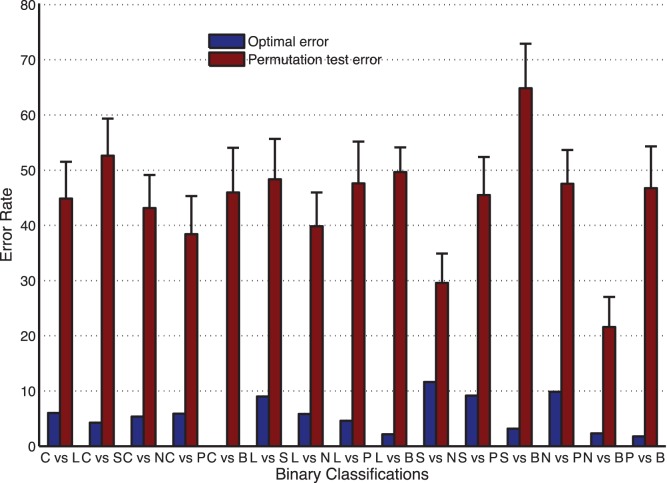
Comparisons of error rate (percentage of misclassified samples) over 50 runs of permutation analysis. The original class memberships of all samples are randomly shuffled for 50 times and then used together with original optimal features for classification using the same cross validation as applied before for original dataset.

Since the relatively small sample size of some subdatasets in the benchmark dataset, it is also important to evaluate the stability and reliability of our classification model. In this paper, permutation tests [72], [73] are performed to compare the misclassification error rates using our model with those from the randomization studies. Initially, the class memberships of all the samples were permuted while keeping features unchanged; then the newly generated random dataset is analyzed by using the same cross validation procedure applied before to the original dataset (SVM parameters are the same as those chosen to obtain the minimum error rates for original datasets). This procedure is also carried out 50 times and the error rates (±standard deviation) over 50 permutations for all binary classifications are shown in Figure 4 and compared with the minimum error rates obtained from original datasets. As one can see, the estimated error rates obtained by our method for original dataset are significantly lower than those from the randomization studies. If we do the same permutation test on the whole original dataset, overall error rate on average is 76.7% (±6.1%), which is much higher than the error rate 21.2% obtained by using optimal features. In summary, classification information can be characterized by optimal features; otherwise, the estimated error rate obtained from original dataset will be close to that calculated from the shuffled dataset.

### Conclusions

In this section, we will summarize our conclusions as follows.

1. From the results on three datasets, our ensemble method is effective and valuable for predicting protein subnuclear localizations compared with existing methods for the same problem.
2. From contribution of features as shown in Table 3 and 6, Combination1 and HHT make the most important contribution, DWT and PPDD the second, and RQA is worst.
3. The method GFO can effectively find the optimal RBF kernel parameter and further speed up our method.
4. This problem cannot be solved by simply using popular machine learning classifiers (such as SVM, RF).
5. The weight strategy is important for this problem (unbalanced dataset).
6. Two-step optimal feature selection method is effective.
7. Effective classification for nuclear speckles and nucleolus is the key factor.

Although our method obtain relatively satisfactory results, some open problems need to be investigated in the future. Subnuclear localization prediction can be considered multi-label, unbalanced problem. Hence, popular methods for multi-label, unbalanced problems may be applied to improve this work.

## Methods

### Feature Extraction Methods Based on Amino Acid Classification

Suppose that 20 amino acids are divided into 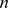 groups, denoted by 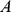, according to certain classification method listed in Table 1. Then, for a given protein sequence 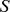 of length 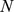, we may obtain a new sequence 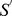 of 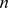 symbols with the same length as 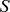, each symbol corresponding to one group of amino acids.

#### Local amino acid composition (LAAC) and local dipeptide composition (LDC)

Protein targeting signals are fragments of amino acid sequences, usually on N-terminal or C-terminal, responsible for directing proteins to their target locations. They are usually located at the N-terminal or C-terminal of a protein sequence [74]. But they are difficult to detect and define signal motifs. Here we compute local amino acid composition and local dipeptide composition on the first 60 amino acids from the N-terminal and 15 amino acids from the C-terminal of a protein sequence to represent protein targeting signals, which is inspired by [11]. Finally, 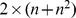 features are generated.

#### Global descriptor (GD)

The global descriptor method was proposed first by [34] for predicting protein folding classes and later applied to predict human Pol II promoter sequences [75] and distinguish coding from non-coding sequences in a prokaryote complete genome [76] by our group. The global descriptor contains three parts: composition (*Comp*), transition (*Tran*) and distribution (*Dist*). *Comp* describes the overall composition of a given symbol in the new symbol sequence. *Tran* characterizes the percentage frequency that amino acids of a particular symbol are followed by a different one. *Dist* measures the chain length within which the first, 25, 50, 75 and 100% of the amino acids of a particular symbol are located [34]. Overall, we get 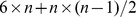 features from the global descriptor for 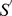.

#### Lempel-Ziv complexity (LZC)

The Lempel-Ziv (LZ) complexity is one of the conditional complexity measures of symbol sequences. It can reflect most adequately the repeated patterns occurring in the symbol sequence and are also easily computed [35]. The LZ complexity has been successfully employed to construct phylogenetic tree [77] and predict protein structural class [78]. Let 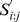 be the subsequence of 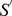 between position 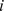 and 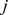. The LZ complexity of sequence 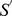, usually denoted by *c*(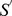), is defined as the minimal number of steps with which *S0* is synthesized from null sequence according to the rule that at each step only two operations are allowed: either copying the longest fragment from the part of 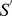 that has already been synthesized or generating an additional symbol. Suppose that the sequence 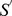 is decomposed into.
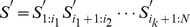
This decomposition is also called the exhaustive history of 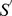, denoted by 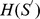. It is proved that every sequence has a unique exhaustive history [35]. For example, for the sequence , its exhaustive history is 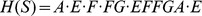, where “

” is used to separate the decomposition components. So, 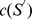 = 6.

### Feature Extraction Methods Based on Physicochemical Properties

#### Autocorrelation descriptors (AD)

Three widely-used autocorrelation descriptors are selected: normalized Moreau-Broto autocorrelation descriptors, Moran autocorrelation descriptors and Geary autocorrelation descriptors [36]. They are all defined based on the value distributions of 30 physicochemical properties of amino acids along a protein sequence (see Table 2). The measurement values of these properties are first standardized to have zero mean and unit standard deviation and then the three autocorrelation descriptors are calculated. These descriptors are also used for the classification of G-protein-coupled receptors by Peng et al. [79].

The *normalized Moreau-Broto autocorrelation descriptors* are defined as.
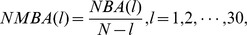
where 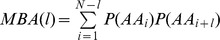, 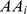 and 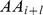 are the amino acids at position 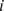 and 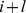 along the protein sequence, respectively. 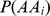 and 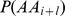 are standardized property values of amino acid 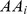
and 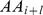, respectively. The maximum value of 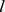 is set at 30 as in [36].

The Moran autocorrelation descriptors are defined as.
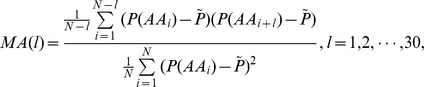
where 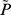 is the mean value of the property under consideration along the sequence.

The Geary autocorrelation descriptors are defined as.
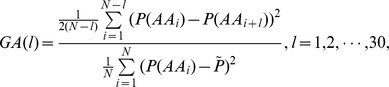



For each AD, we obtain 900 ( = 30×30) features. In total, 2700 ( = 900×3) features are obtained to describe a protein sequence.

#### Sequence-order descriptors (SD)

In order to derive the sequence-order descriptors, we use two distance matrices for amino acid pairs. One is called the *Grantham chemical distance matrix* [36], and the other is called the *Schneider-Wrede physicochemical distance matrix* [37]. Then, the *jth-rank sequence-order coupling number* is defined as.
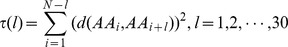
where is 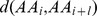 one of the above two distances between two amino acids 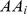 and 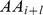 located at position 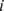 and position 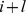, respectively.

The *quasi-sequence-order descriptors* are defined as.
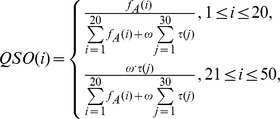
where 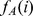 is the occurrence frequencies of 20 amino acids in a protein sequence and 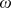 is a weighting factor (with default 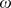 = 0.1).

We end up with 60 ( = 30×2) sequence-order-coupling numbers and 100 ( = 50×2) quasi-sequence-order descriptors. In total, there are 160 features extracted from SD.

#### Auto covariance (AC)

The autocovariance method is a statistical tool proposed by Wold *et al.* [38] which can capture local sequence-order information. It has been applied to many fields of bioinformatics, such as functional discrimination of membrane proteins [39], predicting protein submitochondria locations [40], and so on.

The *autocovariance method* is defined as.
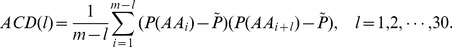
The AC is computed on 531 physicochemical properties mentioned earlier in Subsection Physicochemical properties.

#### Physicochemical property distribution descriptor (PPDD)

The physicochemical property distribution descriptor is first proposed by [41] for remote homology detection. In this descriptor, the protein sequence of length 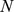 is first transformed from the 20 amino acid letter code to 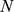-dimension numerical vector associated with the index being used. The average across all 4-mers is taken to create a new 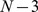-dimensional numerical vector. This new vector is then normalized to have the mean and standard deviation of the theoretical values associated with the index. This normalized numerical vector is transformed into a discrete distribution of 18 frequency values, where each value represents a range of 0.5, i.e., the first bin contains all values less than −4, the second bin contains all values between −4 and −3.5, and so forth. So, for every physicochemical property, the physicochemical property distribution descriptor generates 18 features.

#### Recurrence plot and recurrence quantification analysis (RQA)

Recurrence plot (RP) is a purely graphical tool originally proposed by [80] to visualize patterns of recurrence in the data. A time series 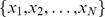 with length 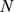 can be embedded in the space 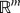 with embedding dimension 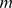 and a time delay 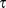 according to nonlinear dynamic theory [81]. Supposing that 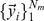 represents a trajectory in the corresponding phase space, we have.
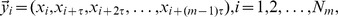
where 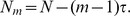
Once a norm function has been selected (e.g., the commonly chosen Euclidean norm [82]), we can calculate the distance matrix (DM) from the above points 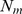. DM is an 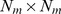 square matrix whose elements are the distances between any pair of points. DM can be transformed into a rescaled distance matrix (RDM) through dividing each element in the DM by the maximum value of DM [81]. After obtaining RDM, it can be further transformed into a recurrence matrix (RM) whose elements are 0 or 1 by choosing a threshold 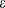. The elements of RM are calculated by the following equation:



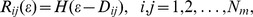
Where 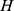 is the Heaviside function



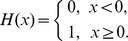
RP is obtained by visualizing RM using different colors for its binary elements (e.g., plotting a black dot at the coordinates 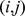, if 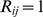 and a white dot, if 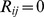). For any 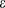, since 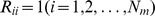 by definition, the RP always has a black main diagonal line. Furthermore, the RP is symmetric with respect to the main diagonal as 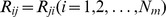. An important step is to choose the parameter 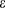 of RP. If 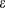 is not chosen appropriately, we would not extract sufficient information about the underlying system [83], [84]. In this paper, we use an approach similar to [85] to fix the embedding dimension 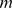, time delay 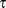 and radius 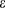. Finally, we set the embedding dimension *m = *4, time delay 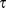 *= *1, and the radius 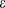 *= *20% of the mean Euclidian distance between points.

In order to overcome physical limitation of RP, [42] proposed a new nonlinear technique, namely the recurrence quantification analysis (RQA), to quantify the information in the RP based on diagonal structures and vertical structures. In recent years, RQA has been successfully applied in many different fields [39], [42], [82], [84]–[88]. Now there are 14 recurrence variables developed to quantify RP [81], [83]. The definitions of these 14 recurrence variables are omitted here due to the page limit. A detailed description of these recurrence variables can be found in [81], [83] and the references therein.

#### Discrete wavelet transform (DWT)

It is known that low-frequency internal motions do exist in protein and DNA molecules and indeed play a significant role in biological functions [89]. And DWT can elucidate simultaneously both spectral and temporal information and is particularly helpful in detecting subtle time localized changes [43]. So, DWT can be used to reflect the order effect of a protein sequence. DWT decomposes the signal into coefficients at different scales. The coefficients of the DWT contain the approximation coefficient, which represents the high-scale and low-frequency components of the signal, and the detail coefficient, which represents the low-scale and high-frequency components of the signal [90]. We apply DWT on the converted numerical signal of the protein sequence by using the selected 531 indices. Suppose that every signal is decomposed into 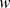 scales with details from scale 1 to scale 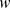 and an approximation at scale 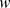 by the DWT, and the wavelet coefficients of (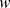
+1) scales are obtained in total. To convert the wavelet coefficients into a feature vector with fixed length better suited for machine learning’s algorithms, the statistics over the set of wavelet coefficients are used as in [91]. The following statistical features extracted from the approximation coefficients and detail coefficients are used: (i) maximum of the wavelet coefficients at each scale, (ii) mean of the wavelet coefficients at each scale, (iii) minimum of the wavelet coefficients at each scale, and (iv) standard deviation of the wavelet coefficients at each scale. So for every index, a protein sequence can be represented as a 4(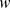
+1) -dimensional feature vector. In this study, the Bior3.1 wavelet function was selected as the appropriate wavelet function and the decomposition level 5 was chosen [92].

#### Hilbert-Huang transform (HHT)

The HHT consists of two parts: empirical mode decomposition (EMD) and Hilbert spectral analysis (HSA). EMD is a time-frequency analysis method and was originally proposed by [44] for the study of ocean waves. Similar to other time-frequency methods, such as Fourier analysis and wavelet analysis, EMD adaptively decomposes a time series into a definite set of intrinsic mode functions (IMFs) by means of an algorithm called sifting process [44]. However, the base functions of Fourier and wavelet analyses are pre-determined and are not suitable for nonlinear systems [44]. The detailed information about IMFs and sifting process can be found in [44]. After sifting, the original signal can be reconstructed as.
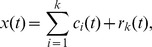
where 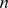 is the number of IMF, 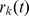 denotes the final residual, and 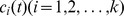 is an IMF that is nearly orthogonal to each other. The EMD method also has been used by our group to simulate geomagnetic field data [93] and predict the subcellular localizations of apoptosis proteins recently [86]. In order to convert IMFs into a feature vector with fixed length better suited for classifiers, we propose to use the following statistical features extracted from IMFs: (i) maximum of every IMF, (ii) mean of every IMF, (iii) minimum of every IMF and (iv) standard deviation of every IMF.

After EMD, we apply the Hilbert transform to each IMF component and obtain an analytic signal:
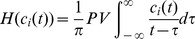


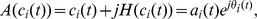
where, 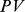 indicates the principle value of the singular integral, 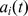 is the instantaneous amplitude, and 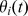 is the phase function:



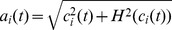


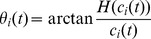
The instantaneous frequency can thus be calculated as.



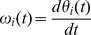
The marginal spectrum 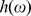 (or the Hilbert-Huang spectrum) can be obtained by integrating with respect to the time variable:



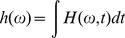
The marginal spectrum offers a measure of total amplitude contribution from each frequency.If the Hilbert-Huang spectrum is denoted as a function of frequency 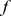 instead of angle frequency 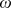, the marginal spectrum can be calculated for each IMF and then normalized by.



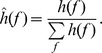
(c.f. [45]). Then, applying the Shannon entropy theory to the normalized marginal spectrum, the Hilbert-Huang spectral entropy (HHSE) is obtained as.



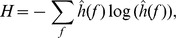


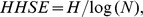
where 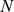 is the number of frequency components and the value of HHSE varies between 0 (complete regularity) and 1 (maximum irregularity).

For each physicochemical property index selected, 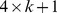 features are obtained in total in HHT.
